# Increasing brain N‐acetylneuraminic acid alleviates hydrocephalus‐induced neurological deficits

**DOI:** 10.1111/cns.14253

**Published:** 2023-05-24

**Authors:** Zhangyang Wang, Xiaoqun Nie, Fang Gao, Yanmin Tang, Yuanyuan Ma, Yiying Zhang, Yanqin Gao, Chen Yang, Jing Ding, Xin Wang

**Affiliations:** ^1^ Department of Neurology, Zhongshan Hospital Fudan University Shanghai China; ^2^ CAS Key Laboratory of Synthetic Biology, CAS Center for Excellence in Molecular Plant Sciences Chinese Academy of Sciences (CAS) Shanghai China; ^3^ Department of the State Key Laboratory of Medical Neurobiology and MOE Frontiers Center for Brain Science, Institutes of Brain Science Fudan University Shanghai China

**Keywords:** hydrocephalus, metabolomics, N‐acetylmannosamine, N‐acetylneuraminic acid

## Abstract

**Aims:**

This metabolomic study aimed to evaluate the role of N‐acetylneuraminic acid (Neu5Ac) in the neurological deficits of normal pressure hydrocephalus (NPH) and its potential therapeutic effect.

**Methods:**

We analyzed the metabolic profiles of NPH using cerebrospinal fluid with multivariate and univariate statistical analyses in a set of 42 NPH patients and 38 controls. We further correlated the levels of differential metabolites with severity‐related clinical parameters, including the normal pressure hydrocephalus grading scale (NPHGS). We then established kaolin‐induced hydrocephalus in mice and treated them using N‐acetylmannosamine (ManNAc), a precursor of Neu5Ac. We examined brain Neu5Ac, astrocyte polarization, demyelination, and neurobehavioral outcomes to explore its therapeutic effect.

**Results:**

Three metabolites were significantly altered in NPH patients. Only decreased Neu5Ac levels were correlated with NPHGS scores. Decreased brain Neu5Ac levels have been observed in hydrocephalic mice. Increasing brain Neu5Ac by ManNAc suppressed the activation of astrocytes and promoted their transition from A1 to A2 polarization. ManNAc also attenuated the periventricular white matter demyelination and improved neurobehavioral outcomes in hydrocephalic mice.

**Conclusion:**

Increasing brain Neu5Ac improved the neurological outcomes associated with the regulation of astrocyte polarization and the suppression of demyelination in hydrocephalic mice, which may be a potential therapeutic strategy for NPH.

## Graphical abstract

Cerebrospinal fluid levels of N‐acetylneuraminic acid (Neu5Ac) decrease in patients with normal pressure hydrocephalus (NPH). Neu5Ac levels are also inversely correlated with severity‐related clinical parameters of NPH. Increasing brain Neu5Ac by N‐acetylmannosamine alleviates the hydrocephalus‐induced neurological deficits in mice, which associates with the modulation of astrocyte polarization and the attenuation of demyelination.

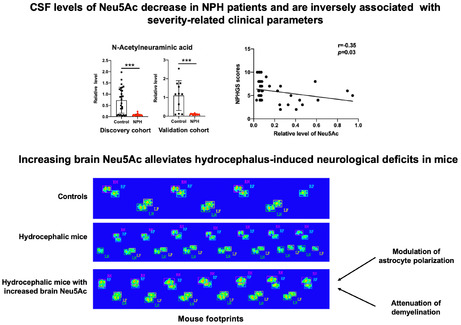

## 1 INTRODUCTION

Normal pressure hydrocephalus (NPH) is the most common type of hydrocephalus in adults over 60‐year‐old,^1^ which can be further classified into idiopathic NPH (iNPH) with unknown etiology and secondary NPH (sNPH) with identified etiologies.^1^, ^2^ The estimated prevalence of NPH is >22 people per 100,000.^3^ According to epidemiological surveys, the incidence rate of NPH increases with age and reaches over 5.9% in the population aged >80 years.^4^ It can cause gait disturbances, dementia, and urinary incontinence.^5^, ^6^ Besides these common neurological symptoms, over 70% of patients with NPH suffer from neuropsychiatric symptoms, including anxiety, which affect the quality of life and increases the stress of caregivers.^7^ Without accurate diagnosis and treatment, these symptoms gradually aggravate and cannot be reversed.^1^ Cerebrospinal fluid (CSF) shunting is the only definitive treatment for NPH.^1^ However, the long‐term efficacy of this procedure remains controversial, particularly for cognitive impairment.^8^ Therefore, a quest for novel treatment strategies is needed and requires exploration of the NPH mechanism.

In NPH, ventricular enlargement caused by increased CSF can cause mechanical stress on the surrounding parenchyma and blood vessels, causing hypoperfusion and hypoxia regionally and globally.^9^, ^10^, ^11^ During this process, the normal metabolism is severely disturbed.^12^, ^13^

Metabolic disturbances could initiate a cascade of serial brain damage, including astrogliosis and demyelination, which eventually leads to clinical symptoms.^14^, ^15^, ^16^ Multiple metabolites have been found to be altered in NPH patients.^17^, ^18^ For example, a reduction in glucose metabolism was observed in iNPH patients.^17^ Increased CSF levels of serine and 2‐hydroxybutyrate and decreased levels of glycerate and N‐acetylneuraminic acid (Neu5Ac) have been reported to distinguish NPH from Alzheimer's disease.^18^ However, to date, the correlation between altered metabolite levels and neurological deficits in NPH has not been well characterized. The lack of experimental validation in vivo also hinders the discovery of novel therapeutic strategies for NPH.

Neu5Ac is the most common sialic acid and the key component at the terminal position of glycoconjugates on the cell surface, such as glycoproteins and glycolipids.^19^ Decreased cellular Neu5Ac caused by genetic mutations led to similar symptoms to NPH, such as cognitive impairment and decreased motor function.^20^ Removing Neu5Ac by neuraminidase could induce hydrocephalus in rats.^21^ These results provided a rationale for evaluating the role of Neu5Ac in NPH.

In the brain, decreased conjugated Neu5Ac can lead to increased infiltration and activation of astrocytes, which is also evident in untreated NPH patients and hydrocephalic mice.^14^, ^22^ In NPH, astrogliosis is believed to decrease intracranial compliance and affect shunt response.^23^ Recent studies have shown that reactive astrocytes exist after brain injuries in the A1 and A2 forms.^24^ A1 astrocytes upregulate multiple complement‐related genes and damage various brain cells, while A2 astrocytes exert neuroprotective effects by releasing neurotrophic factors.^24^, ^25^ Although the polarization of astrocytes is unknown in NPH, promoting the transformation of A1 astrocytes to A2 astrocytes, along with decreasing astrogliosis, has become an emerging pharmacological target in various other neurological disorders.^26^ Therefore, the relationship between Neu5Ac and astrocyte polarization in NPH merits further investigation.

As over 70% of Neu5Ac in the brain constitutes gangliosides, the essential components of myelin,^27^ decreased conjugated Neu5Ac in both humans and mice reportedly resulted in demyelination.^28^, ^29^ In NPH patients and hydrocephalic mice, along with astrogliosis, demyelination has also been reported.^14^, ^22^ Demyelination contributes to the neuropathological and clinical symptoms of NPH^23^. Inhibiting demyelination in hydrocephalus has been proven to be beneficial to cognitive and motor symptoms.^22^ Considering that increasing Neu5Ac upregulates many myelination‐responsive genes, including MBP,^30^, ^31^ the effect of Neu5Ac on the rescue of hydrocephalus‐induced demyelination and related neurological deficits needs to be explored.

In the present study, we explored the metabolic profiling of CSF from patients with NPH and non‐NPH controls. We aimed to clarify the association between differential metabolites and astrocyte polarization, demyelination, and neurological deficits in NPH patients. Our study will help decipher the pathophysiology of NPH and provide a novel strategy for hydrocephalus therapy.

## 2 METHODS

### 2.1 General information

The research protocol was developed in compliance with the Declaration of Helsinki and was approved by the Ethics Committee of Zhongshan Hospital, Shanghai, China (B2018‐062R). Written informed consent was obtained from all the participants.

From March 2018 to January 2019, a senior attending physician recruited participants for the study cohort from Zhongshan Hospital. The NPH group included patients with possible iNPH and sNPH, according to the guideline.^1^ The control group consisted of non‐NPH patients with inflammatory demyelinating diseases who consulted the aforementioned physician and underwent lumbar puncture during the same period when NPH patients were enrolled. Inflammatory demyelinating diseases considered in the enrolment included Guillain‐Barré syndrome (GBS), multiple sclerosis (MS), neuromyelitis optica (NMO), chronic inflammatory demyelinating polyneuropathy (CIDP), and acute transverse myelitis (ATM). Basic demographic characteristics and clinical data were also obtained. The disease severity of NPH was assessed using the normal pressure hydrocephalus grading scale (NPHGS). The Modified Rankin Scale (mRs) was used to monitor neurological disability.

### 2.2 Collection of CSF

CSF was collected by lumbar puncture and immediately transported to the laboratory on ice. The CSF samples were centrifuged at 2000 *g* at room temperature for 10 min. The supernatants were then aliquoted and stored at −80°C until further use.

### 2.3 Metabolomics analysis

As described previously,^32^ metabolites were extracted and analyzed using an ultrahigh‐performance liquid chromatography‐mass spectrometry system (Waters Corporation) coupled to a Q Exactive hybrid quadrupole‐orbitrap mass spectrometer (ThermoFisher). A Luna NH2 column (50 × 2 mm, 5 μm particle size; Phenomenex) was used for chromatographic separation. The mass spectrometer was run in both electrospray ionization positive (ESI^+^) and negative (ESI^−^) modes.

Metabolomics data were processed according to an integral workflow, including quality checks, correction of signal drifts, and peak normalization with XCMS online. Metabolites were identified using accurate mass spectrometry and MS/MS spectra matching the mzCloud database and in‐house spectral libraries. Principal component analysis (PCA) and orthogonal partial least‐squares discriminant analysis (OPLS‐DA) were performed using the SIMCA software (Umetrics AB) to evaluate grouping trends and identify vital metabolites contributing to group separation based on the variable importance in the projection (VIP) values. Multi Experimental Viewer software (DFCA) was used for univariate analysis. Multiple linear regression, a statistical method, was used to adjust for potentially confounding variables (age and sex), similar to previous studies.^33^, ^34^, ^35^ Metabolites with *p* < 0.05, false discovery rate (FDR) <0.05, and VIP values>1.00 were defined as statistically significant. Hierarchical clustering was visualized as heatmaps with the aid of R language. Correlation analysis was performed using the IBM SPSS 22 software (IBM Corp).

### 2.4 Experimental design of the animal study

Adult male wild‐type C57BL/6J mice (12‐week‐old, body weight 29–31 g) were purchased from the Beijing SPF Biotechnology Company. Animals had free access to food and water and were housed in the same controlled environment with a humidity of 55 ± 10%, a temperature of 22 ± 2°C, and 12–12 h light–dark cycle. Experimental procedures were approved by the Ethics Committee of Zhongshan Hospital.

Animal studies were performed according to the Animal Research: Reporting of In Vivo Experiments (ARRIVE) guidelines 2.0 to ensure transparency and reproducibility in the reporting of animal research.^36^, ^37^, ^38^ The sample size for the animal experiments was determined using a pilot experiment. To ensure the random assignment of mice to groups, we used a random number generator. The mice were assigned to one of three groups: Ctr + Veh group, which served as the animal control and received 0.9% saline vehicle; HCP + Veh group, which consisted of hydrocephalic mice receiving 0.9% saline vehicle; and HCP + ManNAc group, which consisted of hydrocephalic mice receiving N‐acetylmannosamine (ManNAc) administration. Researchers were blinded to the group allocation during the whole experiment. After the induction of hydrocephalic mice or animal control, ManNAc (Sigma‐Aldrich) or saline vehicle (0.9% sterile saline) was immediately administered subcutaneously and continued twice daily. According to previous reports, subcutaneous administration of ManNAc could increase its retention time and bioavailability.^39^, ^40^ Regardless of the group, a standard 0.3 mL volume was used for each administration. Based on previous studies on ManNAc administration,^41^, ^42^ 0.5 g/kg ManNAc was administered twice a day to explore its effect on increasing the brain Neu5Ac in hydrocephalic mice. Using the foot‐fault test and glial fibrillary acidic protein (GFAP) immunostaining on day 7, the optimal dose of ManNAc was determined by analyzing the effect of different doses on motor coordination and astrogliosis in hydrocephalic mice, the two core pathological features of NPH. The dose gradient of ManNAc was set as follows: 0.5, 1, 2 g/kg/day, according to previous literatures.^41^, ^42^ A subsequent experimental timeline of the optimal dose of ManNAc is shown in Figure 3A. To minimize confounding factors, different groups were housed together in the same cages and all experiments were conducted at the same time. Furthermore, all outcome assessments were performed in a blinded manner to reduce the potential for bias.

### 2.5 Kaolin‐induced hydrocephalic mice

The mice were anesthetized with 5% isoflurane induction and 1.5% isoflurane maintenance throughout the procedure. The atlantooccipital membrane was aseptically exposed after careful skin incision and muscle separation under a surgical microscope. A 30‐gauge needle attached to a 25 μL micro‐syringe was inserted 1–2 mm into the cisterna magna under the atlantooccipital membrane. Subsequently, 10 μL of 10% kaolin solution in saline was injected using a syringe pump at a speed of 1 μL/min. At the end of the injection, kaolin was allowed to circulate in the CSF for 5 min with the needle indwelling. The needle pinhole was closed with biological glue, and the incision was sutured. The mice were kept warm on a heating pad to maintain the temperature until they fully recovered from anesthesia. For the animal control, 10 μL normal saline was injected.

### 2.6 Brain Neu5Ac measurement

Frozen brain tissue was homogenized in cold PBS and centrifuged at 12000 *g* for 15 min. The supernatant was collected to measure the protein concentration using a bicinchoninic acid (BCA) assay kit (ThermoFisher) and the Neu5Ac content using a colorimetric assay kit (Jiancheng). The Neu5Ac content was expressed as nanomoles per milligram of protein (nmol/mg).

### 2.7 Immunofluorescent staining and quantification

Coronal brain slices (25 μm thick) were used for immunofluorescence staining as described previously.^43^ Slices were incubated with the following primary antibodies: C3d (ab56164, 1:100, Abcam), GFAP (ab209384, 1:4000, Abcam), S100A10 (ab184718, 1:100, Abcam), and MBP (9746, 1:2000, Cell Signaling Technology). Respective Alexa‐Fluor‐conjugated secondary antibodies (ThermoFisher) were subsequently used. Images were captured using an FV1000 confocal microscope (Olympus Corporation). The percentage of C3d^+^ GFAP^+^ and S100A10^+^ GFAP^+^ cells on the lateral side of the PVW was calculated. Image *J* software (NIH, Bethesda) was used to measure the mean MBP fluorescence intensity and GFAP‐positive area (%) in the PVM.

### 2.8 Western blots

The PVW of each animal was separated. Total protein was extracted using RIPA Lysis buffer mixed with Protease Inhibitor Cocktail (ThermoFisher) and quantified using a BCA protein assay kit (ThermoFisher). Western blotting was performed as previously described.^43^ Primary antibodies, including C3d (ab56164, 1:1000, Abcam), GFAP (ab209384, 1:1000, Abcam), S100A10 (ab184718, 1:1000, Abcam), and GAPDH (9746, 1:1000, Cell Signaling Technology), were used. Signals were detected using the enhanced chemiluminescence reagent (Millipore Sigma) with the Bio‐Rad ChemiDoc system (Bio‐Rad), and the results were visualized with Image Lab software (Bio‐Rad).

### 2.9 Mice MRI examination

The mice were anesthetized with 5% isoflurane induction and 1.5% isoflurane maintenance throughout the procedure. MRI examinations were performed using an 11.7 T Bruker Biospec scanner (Bruker Corporation, Ettlingen, Germany). T2‐weighted images were acquired in the coronal plane with a slice thickness of 500 μm (TR/TE =3000/30 ms). After scanning, Image *J* software (NIH) was used for analysis. The Evans' ratio was measured by dividing the maximum width between the lateral ventricles by the maximum width of the whole brain in this particular plane. The area of periventricular white matter (PVM) hyperintensities in the most evident coronal plane were further evaluated.

### 2.10 Luxol fast blue (LFB) staining

Coronal brain slices (25 μm thick) were immersed in 0.1% LFB solution (Cat. S3382; Sigma‐Aldrich) at 60°C for 2 h. The brain slices were then differentiated with 0.05% lithium carbonate (Cat. no. 255823; Sigma‐Aldrich) for 30 s and rinsed in 70% ethanol until the white matter was visualized. Finally, the slices were washed with distilled water, dehydrated, and fixed. Images were obtained using an Olympus AX 70 microscope (Olympus). The intensity of LFB staining was analyzed using the Image *J* software (NIH).

### 2.11 Behavioral test

The rotarod test, foot‐fault test, CatWalk gait analysis, novel object recognition test, and open‐field test were performed according to a previously described protocols.^43^, ^44^, ^45^, ^46^ The balance ability of mice was assessed using a rotarod apparatus (Ugo Basile). The average latency time of three trials to fall off the rotating rod was recorded. Motor coordination of the mice was assessed using the foot‐fault test. The foot‐fault rate of the forelimbs was calculated by dividing the fault steps by the total number of steps in the last minute of movement. The CatWalk system (Noldus) was used for gait analysis. According to the characteristics of specific gait disturbances in NPH patients,^47^ the parameters of speed, step length, step cycle, duty cycle, and diagonal support were analyzed in our study. In addition, a novel object recognition (NOR) test and an open field test were performed. The discrimination index (DI) of the NOR test was calculated using the following formula:

$$\mathrm{DI}=\frac{\left(\text{time exploring the side with novel object}-\text{time exploring the side with familiar object}\right)}{\left(\text{time exploring the side with novel object}+\text{time exploring the side with familiar object}\right)}\times 100\%$$

The preference index (PI) to reflect the preference of the new object over the familiar object was determined using the formula PI=DI_test_‐DI_training_. The percentage of time spent in the central zone for the open field test was calculated and served as a marker of anxiety‐like behavior.

### 2.12 Statistical analysis

The sample size for the clinical research was determined according to the formula used in the cross‐sectional study.^48^ Quantitative data are presented as mean ± SD. Normal distribution and homogeneity of variance were assessed using Shapiro–Wilk and Levene's tests. Differences between groups were analyzed using Student's *t*‐test and one‐way analysis of variance (ANOVA) followed by a post hoc Tukey–Kramer test for multiple comparisons. Differences across groups with time‐related repeated measurements were analyzed using repeated‐measures ANOVA with a post hoc Tukey–Kramer test. In addition, non‐parametric tests were used if necessary. For qualitative data, differences between groups were evaluated using the chi‐square test or Fisher's exact test. *p* < 0.05 was considered statistically significant in all tests.

## 3 RESULTS

### 3.1 General information on NPH patients

**FIGURE 1:**
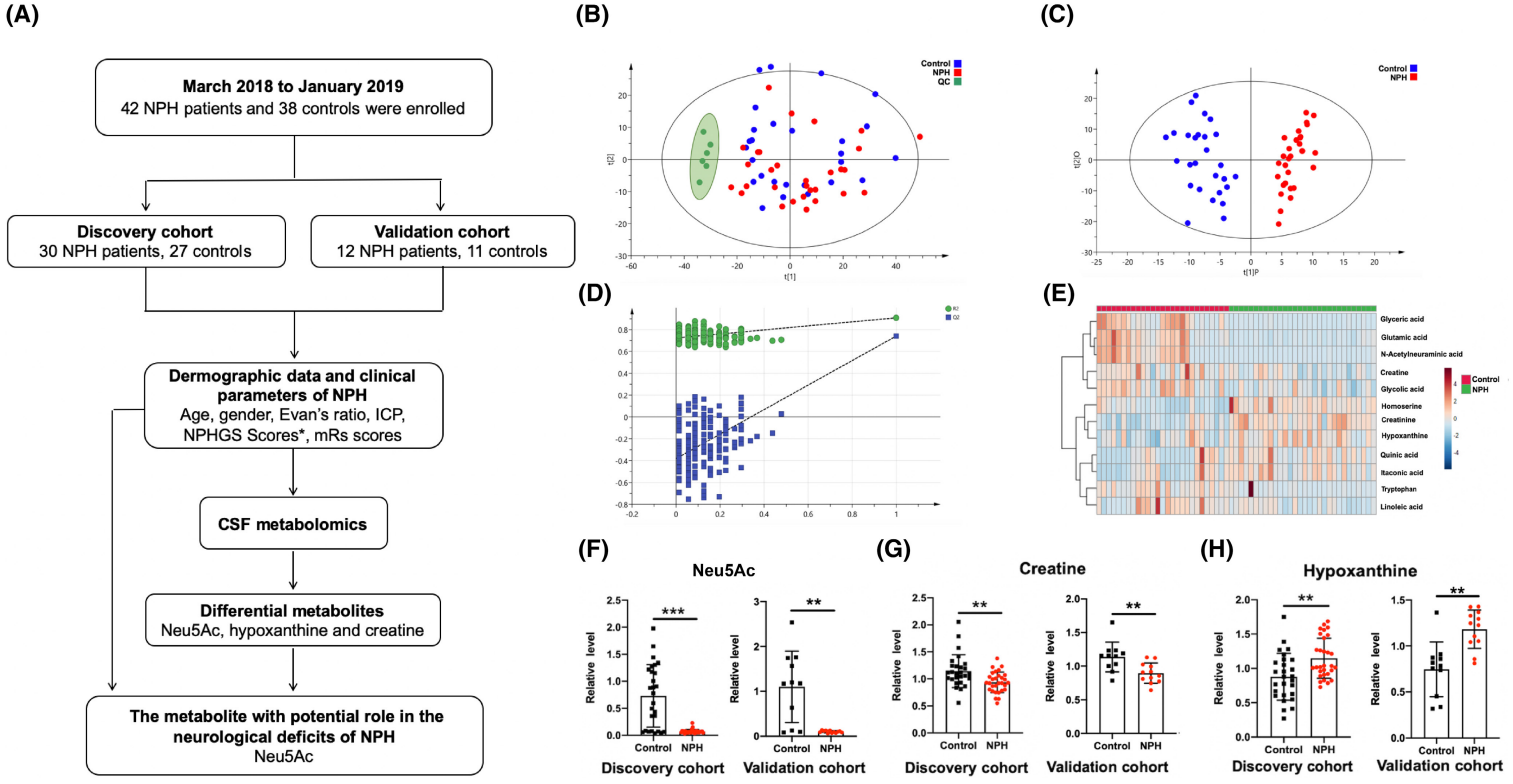
CSF metabolomic analysis in the NPH and control groups. (A) A flow chart of the metabolomic study. * NPHGS scores include assessment of the gait, cognitive and urinary symptoms. ICP: intracranial pressure; NPHGS: normal pressure hydrocephalus grading scale; mRs: Modified Rankin Scale. Neu5Ac: N‐acetylneuraminic acid. (B) PCA score plot between control (*blue*, Control), normal pressure hydrocephalus (*red*, NPH), and quality‐control (*green*, QC) groups in the discovery cohort. (C) OPLS‐DA score plot between control (*blue*) and NPH (*red*) groups in the discovery cohort. (D) Validation plot for OPLS‐DA model obtained from 200 times the permutation test. (E) Heatmap of the 12 differential metabolites in the discovery cohort. Columns represent the samples (control, *red*; NPH, *green*). Rows represent 12 differential metabolites. The colors represent the relative levels of metabolites, changing from blue to red to indicate elevating levels. (F–H) Levels of Neu5Ac (F), creatine (G), and hypoxanthine (H) in the discovery and validation cohorts. Data were evaluated using Mann–Whitney test with the FDR adjustment. ***FDR < 0.001; **FDR < 0.01 Control vs. NPH. For B‐E, *n* = 27 for Control; *n* = 30 for NPH; *n* = 6 for QC. For F‐H, *n* = 27 for Control in the discovery cohort and *n* = 11 in the validation cohort; *n* = 30 for NPH in the discovery cohort and *n* = 12 in the validation cohort. All data were presented as mean ± SD.

A flow chart of the metabolomics study is shown in Figure 1A. Eighty participants were enrolled in our study, including 42 patients with NPH and 38 healthy controls. The NPH group consisted of 33 patients with possible iNPH and 9 patients with sNPH (Table S1). There was no statistical difference in terms of the demographic and clinical parameters between the iNPH and sNPH patients. The control group comprised 38 patients, including 10 with GBS, 8 with MS, 13 with NMO, 5 with CIDP, and 2 with ATM. Eighty participants from the NPH and control groups were randomly divided into discovery and validation cohorts for metabolomic analysis. The demographic and clinical characteristics of the two cohorts are shown in Table 1. The discovery cohort consisted of 30 patients with NPH and 27 healthy controls. The validation cohort was comprised of 12 patients with NPH and 11 controls. In both the discovery and validation cohorts, NPH patients were older and had higher Evans ratios (*p* < 0.001). In the discovery cohort, male patients were more frequently seen with NPH (*p* < 0.01).

**TABLE 1:** Demographics and clinical characteristics of the discovery and validation cohort.

	NPH	Control	*p* Value
Discovery cohort^a^	*n* = 30	*n* = 27	
Age^b^	70.7 ± 11.2	40 ± 13.3	<0.001
Gender (Female/Male)^a^	6/24	18/9	<0.01
Evans' Ratio^b^	0.34 ± 0.03	0.26 ± 0.02	<0.001
NPHGS Scores^b^	6.6 ± 2	N/A	
mRs Scores^b^	2.7 ± 1	N/A	
ICP (mmH_2_O)^b^	145 ± 34.5	141 ± 55	0.74
Validation cohort^a^	*n* = 12	*n* = 11	
Age^b^	73.5 ± 6.4	47.6 ± 10.9	<0.001
Gender (Female/Male)^a^	4/8	7/4	0.15
Evans’ Ratio^b^	0.3 ± 0.03	0.3 ± 0.02	<0.001
NPHGS Scores^b^	4.4 ± 1.83	N/A	
mRs Scores^b^	2.1 ± 0.9	N/A	
ICP (mmH_2_O)^b^	126.8 ± 34.7	143.2 ± 32.6	0.26

Abbreviations: ICP, Intracranial pressure; mRs: Modified Rankin Scale; N/A, Not applicable; NPH, Normal pressure hydrocephalus; NPHGS, Normal pressure hydrocephalus grading scale.

^a^ Categorical variables are expressed as *n*.

^b^ Continuous variables are expressed as median ± SD.

### 3.2 Metabolites are altered in NPH patients

CSF metabolic profiling was performed in the discovery cohort. A total of 2701 metabolite peaks were detected by mass spectrometry, including 1134 and 1567 in ESI^+^ and ESI^−^ modes, respectively. PCA model revealed that the quality‐control samples clustered tightly in the score plot (Figure 1B), indicating the good analytical reproducibility of this metabolomics analysis. OPLS‐DA model revealed that metabolic profiles varied significantly between the two groups. A clearly distinguished separation between NPH patients and controls was observed (R_2_Y = 0.91, Q_2_ = 0.70, Figure 1C), and its validity was confirmed by permutation tests (Figure 1D). We further identified 12 differential metabolites (*p* < 0.05, FDR < 0.05, and VIP > 1.00) between the two groups in the discovery cohort (Table S2). Figure 1E shows that these 12 differential metabolites were clustered in a heatmap.

These 12 metabolites were further evaluated in a validation cohort. As shown in Table 2, only three metabolites, N‐acetylneuraminic acid, hypoxanthine, and creatine, were found to differ significantly in both cohorts after adjustment for age and sex. Hypoxanthine levels were markedly elevated in the CSF samples of NPH patients compared to healthy controls, whereas Neu5Ac and creatine levels had decreased in the CSF of NPH patients (Figure 1F–H, FDR < 0.01). Among these, the fold‐change in Neu5Ac between the two groups was the highest in both the discovery and validation cohorts. It also had the highest VIP and lowest FDR, the highest adjusted R2 value, and the lowest *p* value. Therefore, Neu5Ac was the best metabolic factor to differentiate NPH patients in the discovery and validation cohorts using multivariate or univariate analysis.

**TABLE 2:** Differential metabolites between NPH patients and controls in the discovery and validation cohorts.

Metabolite	*p*		FDR		VIP		Log2 fold‐change^a^		Adjusted *R* ^2^ ^b^	β^b^	*p* ^b^
	*DC*	*VC*	*DC*	*VC*	*DC*	*VC*	*DC*	*VC*			
N‐acetylneuraminic acid	8.23 E‐06	2.77 E‐04	2.79 E‐04	0.001	2.27	1.94	−3.34	−3.42	0.49	−0.58	<0.001
Hypoxanthine	4.02 E‐03	5.07 E‐04	0.03	0.002	1.11	1.47	0.39	0.62	0.22	0.42	0.02
Creatine	2.52 E‐03	5.74 E‐03	0.02	0.006	1.54	1.45	−0.29	−0.32	0.17	−0.49	0.01

Abbreviations: DC, discovery cohort; FDR, false discovery rate; VC, validation cohort; VIP, variable importance in projection;

^a^ Log2 fold‐change: NPH VS Controls.

^b^ Multiple linear regression analysis adjusting for age and gender, variable: NPH‐Controls.

### 3.3 Neu5Ac reduction parallels to severity‐related clinical parameters in NPH patients

**FIGURE 2:**
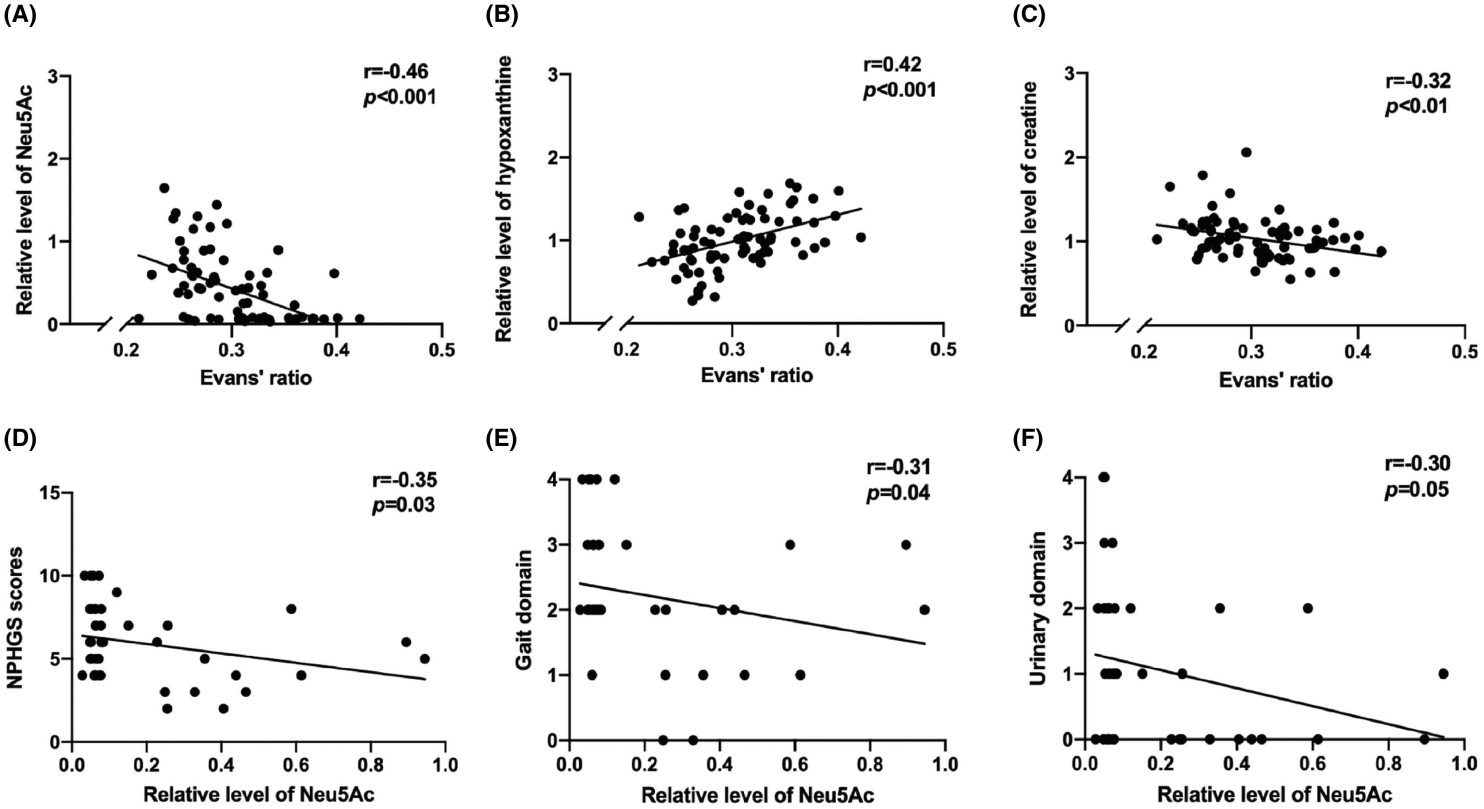
Correlation analysis of the differential metabolites with clinical parameters in NPH patients. (A–C) Correlation of Evans' ratio with relative levels of Neu5Ac (A), creatine (B), and hypoxanthine (C) in the control and NPH groups. For A–B, the Spearman correlation analysis was performed. For C, the Pearson correlation analysis was performed. r: correlation coefficient. (D–F) Correlation of Neu5Ac levels in the NPH group with the total score of NPHGS (D), the NPHGS scores of gait (E), and urinary domain (F). For D–F, the Spearman correlation analysis was performed. For A–C, *n* = 80. For D‐F, *n* = 42. All data were presented as mean ± SD.

We then evaluated the association between these altered metabolites and the clinical parameters of NPH. The correlation analysis results are presented in Table S3. We found that these metabolites were correlated with Evan's ratio (Figure 2A–C, *p* < 0.01), suggesting that metabolic disturbance was associated with ventricle enlargement. Furthermore, there were inverse associations between Neu5Ac levels and NPHGS Scores (Figure 2D, *p* = 0.03) as well as mRs scores (*p* = 0.07, Table S3). This result demonstrated that Neu5Ac is associated with the overall severity of the clinical symptoms of NPH. Meanwhile, the NPHGS scores of the gait and urinary domains were negatively associated with Neu5Ac levels (*p* = 0.04, *p* = 0.05, Figure 2E,F), indicating the potential contribution of decreased Neu5Ac to gait and urinary disturbance in NPH patients. In contrast, hypoxanthine and creatine levels did not correlate with severity‐related clinical parameters (Table S3).

### 3.4 Neu5Ac reduction is reversed in hydrocephalic mice by ManNAc administration

**FIGURE 3:**
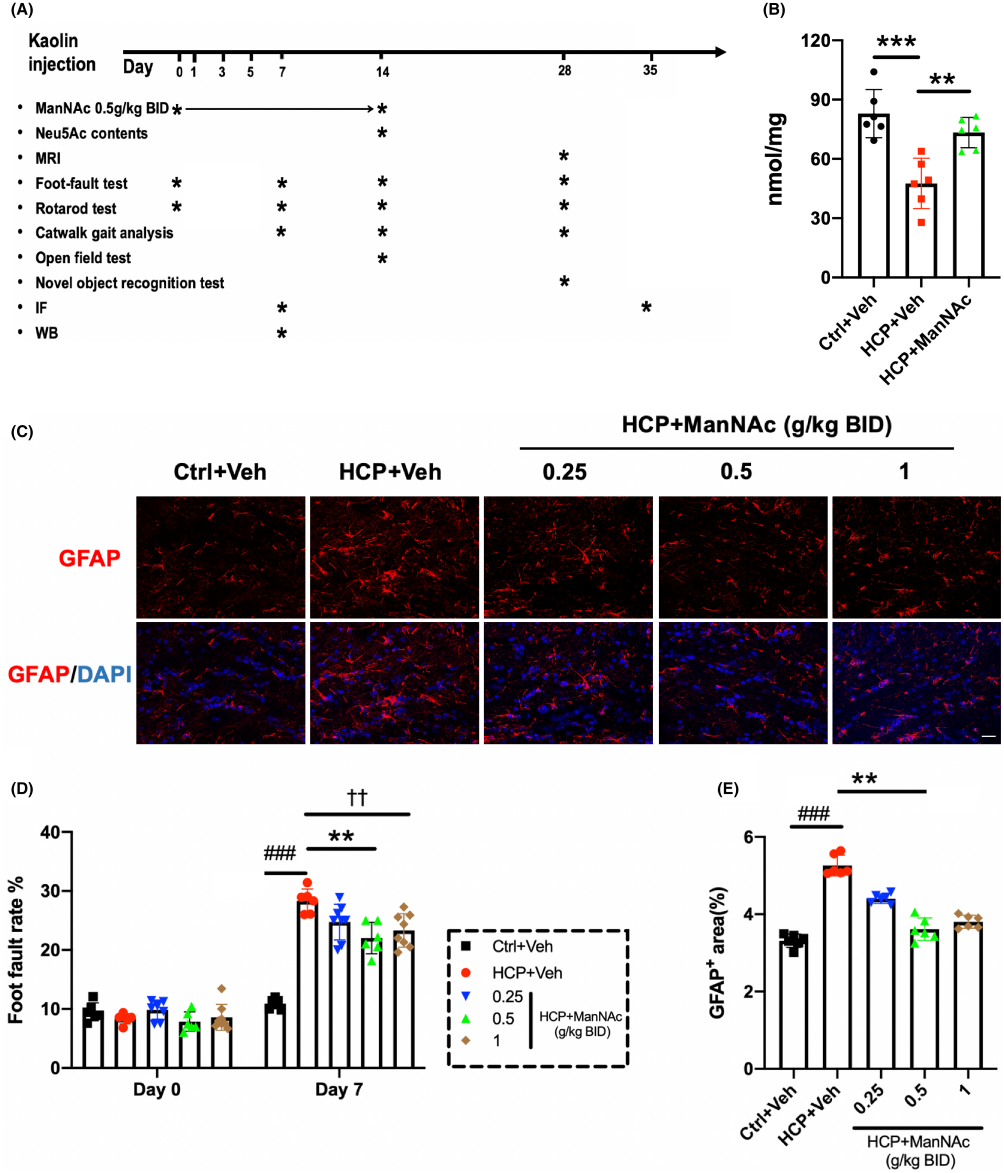
The study design, brain Neu5Ac measurement, and dose gradient of the animal study. (A) Illustration of experimental timeline. Kaolin was injected at day 0, and ManNAc was subcutaneously administrated for 14 days. * represents the timepoint of each experimental technique. IF: immunofluorescence; WB: Western blot; BID: twice a day. (B) Brain Neu5Ac levels in different groups. Data were compared using one‐way ANOVA with the post hoc Tukey–Kramer test. *n* = 6 for Ctrl+Veh, HCP + Veh and HCP + ManNAc, ***p < 0.001; **p < 0.01. (C–E) The effects of various doses of ManNAc on experimental hydrocephalus were assessed by immunostaining of GFAP showing astrogliosis and foot‐fault test showing motor coordination. (C) Representative immunostaining of GFAP (*red*) with DAPI (*Cyan*) in the region of periventricular white matter (scale bar =20 μm). (D) The foot fault rates were assessed using the foot‐fault test. Data were compared by one‐way ANOVA with the post hoc Tukey–Kramer test. (E) Percentage of the GFAP^+^ area in the periventricular white matter. Data were compared using the Kruskal–Wallis with post hoc Dunn test. For C and E, *n* = 6 for HCP + Veh, Ctrl+Veh, 0.25, 0.5, and 1. For D, *n* = 6 for HCP + Veh, Ctrl+Veh, and 0.5, *n* = 8 for 0.25 and 1. For C–E, ###*p <* 0.001 Ctrl+Veh vs. HCP + Veh; **p < 0.01 HCP + Veh vs. 0.5; ^††^ *p <* 0.01 HCP + Veh vs. 1. Ctrl+Veh: animal control + saline vehicle; HCP + Veh: hydrocephalic mice + saline vehicle; 0.25: hydrocephalic mice + ManNAc treatment (0.25 g/kg BID); 0.5: hydrocephalic mice + ManNAc treatment (0.5 g/kg BID); 1: hydrocephalic mice + ManNAc treatment (1 g/kg BID). All data were presented as mean ± SD.

We established a kaolin‐induced hydrocephalic mouse model (HCP) to mimic the NPH patient model^49^ and investigated the neuroprotective effects of Neu5Ac (Figure 3A). First, we explored the alteration of Neu5Ac in hydrocephalic mice and studied whether the exogenous Neu5Ac precursor ManNAc could increase brain Neu5Ac levels (Figure 3B). According to previous studies on ManNAc administration,^41^, ^42^ 0.5 g/kg ManNAc was administered twice a day by subcutaneous injection into a hydrocephalic model (HCP + ManNAc). Hydrocephalic mice with 0.9% saline vehicle were used for comparison (HCP + Veh). Sham mice injected with saline were set as controls (Ctrl+Veh). On day 14, the brain Neu5Ac level in the HCP + Veh group was lower than in the Ctrl+Veh (Figure 3B, *p* < 0.001). After ManNAc administration, the brain Neu5Ac levels in hydrocephalic mice increased significantly (Figure 3B, *p* < 0.01). These results demonstrated that the decreased brain Neu5Ac levels in hydrocephalic mice could be reversed by ManNAc administration.

Next, we explored the optimal therapeutic dose of ManNAc using the hydrocephalic model. It was found that both increased foot‐fault rate and GFAP‐positive area in the HCP + Veh group could be significantly alleviated in the 0.5 g/kg ManNAc‐treated group (Figure 3C–E, *p* < 0.01) on day 7. There was no significant difference between the HCP + Veh group and the 0.25 g/kg ManNAc‐treated group (Figure 3C–E). Concurrently, 1 g/kg ManNAc administration markedly decreased the foot‐fault rate in hydrocephalic mice (Figure 3B, *p* < 0.01) but failed to reduce the GFAP‐positive area (Figure 3C,E). These results confirmed that 0.5 g/kg was the optimal dose of ManNAc in this study, hereinafter referred to as the HCP + ManNAc group.

### 3.5 Increasing Neu5Ac by ManNAc administration modulates the astrogliosis and astrocyte polarization in hydrocephalic mice

**FIGURE 4:**
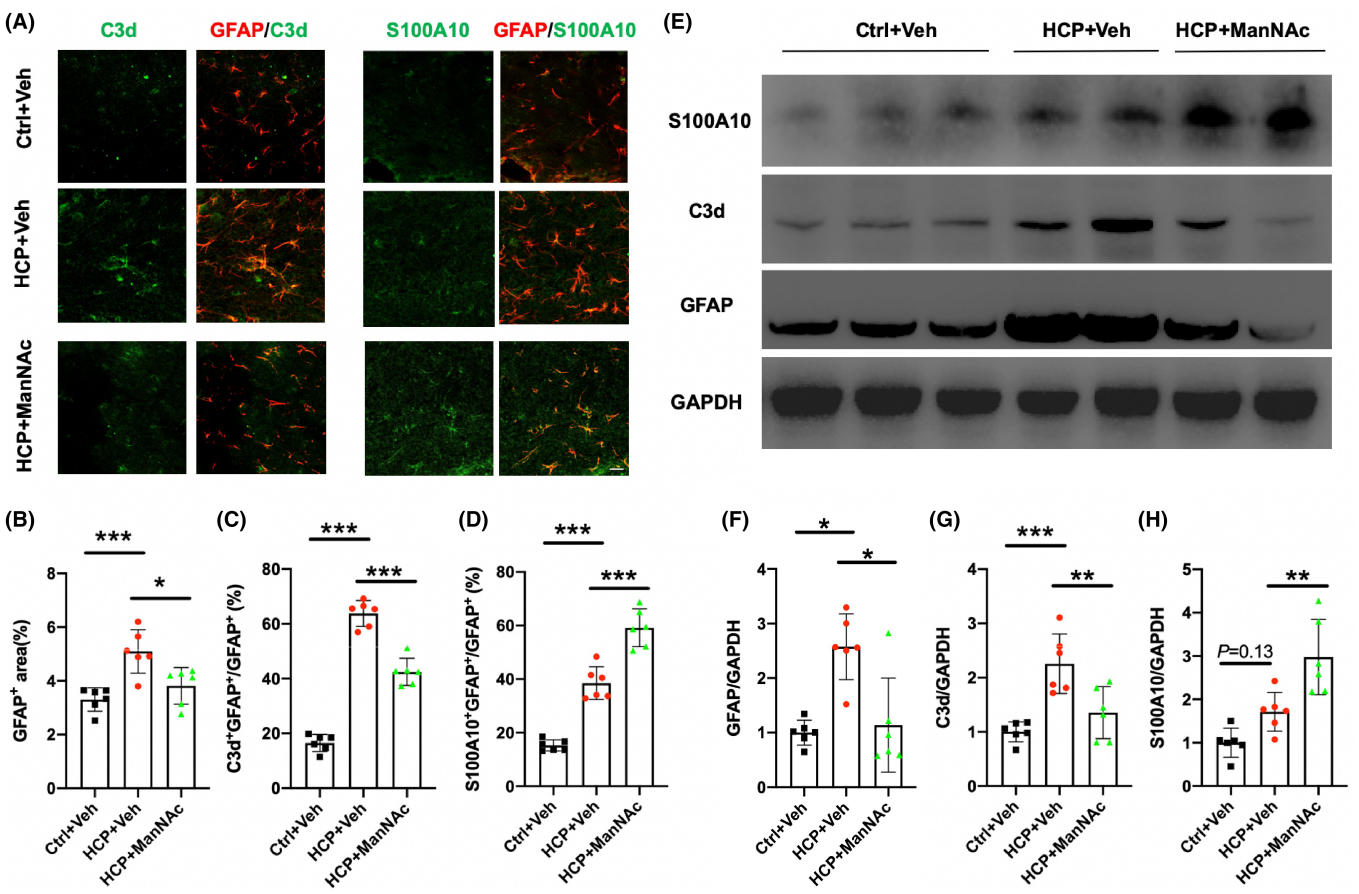
Increasing brain Neu5Ac modulates astrogliosis and astrocyte polarization in hydrocephalic mice. (A) Representative double immunostaining of GFAP (*red*) with C3d (*green*) and GFAP (*red*) with S100A10 (*green*) in the region of periventricular white matter on day 7 (scale bar =20 μm). (B‐D) Percentage of the GFAP^+^ area (B), C3d^+^ GFAP^+^/ GFAP^+^ cells (C), and S100A10^+^ GFAP^+^/ GFAP^+^ cells (D) in the periventricular white matter. Data were compared by one‐way ANOVA with the post hoc Tukey–Kramer test. (E) Representative image of a western blot for GFAP, C3d, and S100A10 in the region of periventricular white matter on day 7. GAPDH was used as the loading control. (F–H) Semi‐quantitative analyses of GFAP (F), C3d (G), and S100A10 (H) expression by western blot in the region of periventricular white matter on day 7. For F and H, data were compared by Kruskal–Wallis with the post hoc Dunn test. For G, data were compared by one‐way ANOVA with the post hoc Tukey–Kramer test. For A‐H, *n* = 6 for Ctrl+Veh: animal control + saline vehicle; HCP + Veh: hydrocephalic mice + saline vehicle; HCP + ManNAc: hydrocephalic mice + ManNAc treatment. ***p < 0.001; **p < 0.01; *p < 0.05. All data were presented as mean ± SD.

We conducted immunofluorescence and immunoblotting in the PVW to evaluate the changes in astrocytes on day 7 (Figure 4A,E). We found an expanded GFAP‐positive area and elevated levels of GFAP expression in the HCP + Veh group (Figure 4B,F; *p* < 0.05), suggesting astrogliosis in hydrocephalic mice. Astrocyte activation was blocked by ManNAc (Figure 4B,F; *p* < 0.05).

Recently, reactive astrocytes have been divided into the A1 and A2 phenotypes.^26^ In hydrocephalic mice, the proportion of C3d^+^ GFAP^+^ cells and C3d expression increased (Figure 4C,G, *p* < 0.001), suggesting A1 polarization. This phenomenon was attenuated following ManNAc administration, which decreased the proportion of C3d^+^ GFAP^+^ cells and C3d expression (Figure 4C,G; *p* < 0.01). In contrast, ManNAc increased the proportion of S100A10^+^ GFAP^+^ cells and S100A10 expression in hydrocephalic mice, suggesting the promotion of A2 polarization (Figure 4D,H, *p* < 0.01). Considered together, these findings suggest that increased brain Neu5Ac inhibited astrocyte activation and promoted the transition from A1 to A2 astrocytes.

### 3.6 Increasing Neu5Ac by ManNAc administration alleviates demyelination in hydrocephalic mice

**FIGURE 5:**
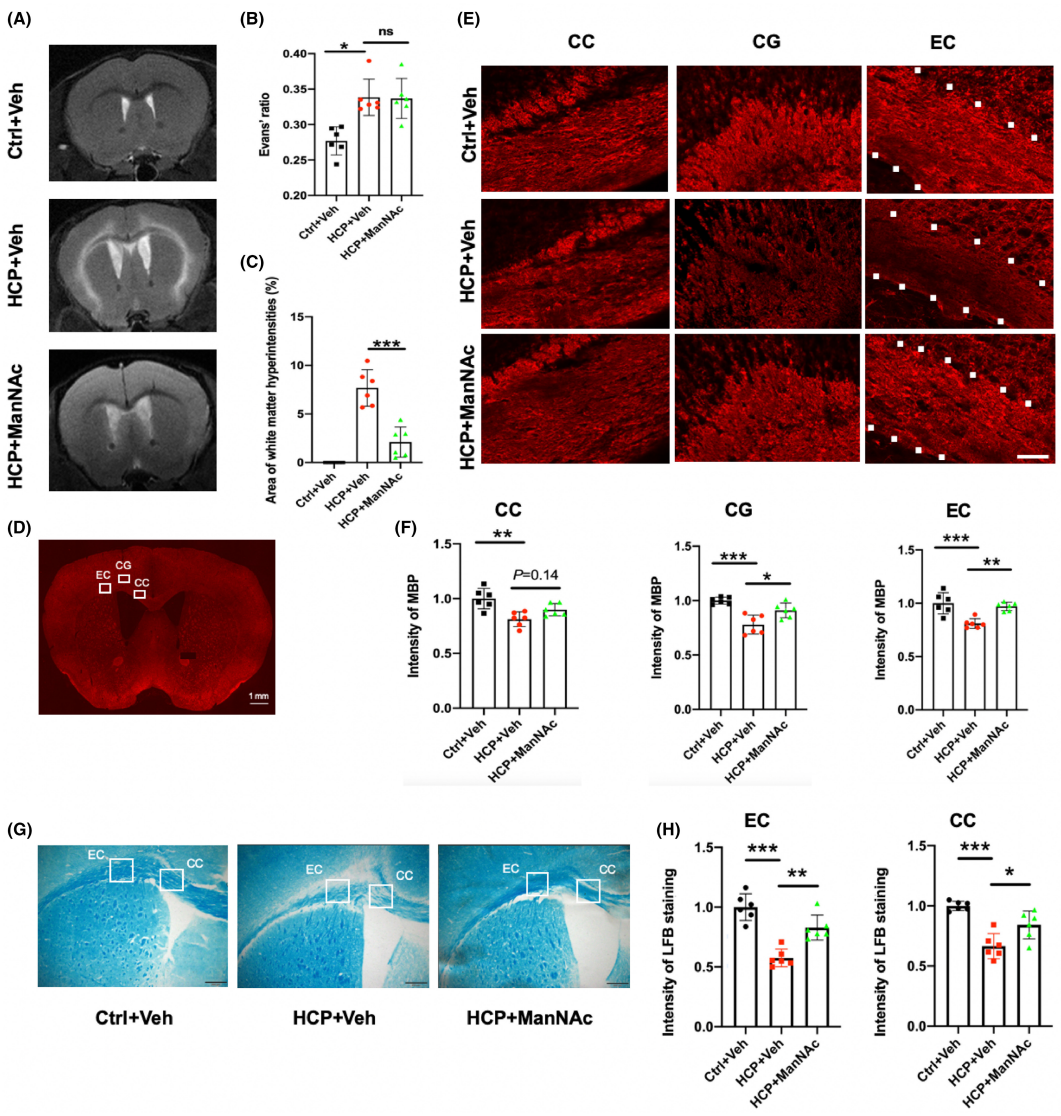
Increasing brain Neu5Ac reduces the white matter damage in hydrocephalic mice. (A) Representative T2‐weighted MRI images in the coronal plane on day 28. (B‐C) Evans' ratio (B) and area of white matter intensities (C) on day 28. For B, data were compared by Kruskal–Wallis with the post hoc Dunn test. For C, data were compared by unpaired t‐test. (D) Illustration of the positions of CC, CG, and EC in an immunofluorescent image of MBP staining (scale bar = 1 mm). (E) Representative immunostaining of MBP in regions of corpus callosum (CC), cingulum bundle (CG), and external capsule (EC) on day 35. The dashed line depicts the border of the EC region (scale bar = 40 μm). (F) Relative quantification of the MBP fluorescence intensity in CC, CG, and EC regions. Data were compared by one‐way ANOVA with the post hoc Tukey–Kramer test. (G) Representative LFB staining in regions of CC and EC on day 35. (H) Relative quantification of the LFB staining intensity in EC and CC region (scale bar = 200 μm). Data were compared by one‐way ANOVA with the post hoc Tukey–Kramer test. For A‐H, *n* = 6 for Ctrl+Veh: animal control + saline vehicle; HCP + Veh: hydrocephalic mice + saline vehicle; HCP + ManNAc: hydrocephalic mice + ManNAc treatment. ***p < 0.001; **p < 0.01; *p < 0.05. ns, not significant. All data were presented as mean ± SD.

In patients with NPH, white matter lesions adjacent to enlarged ventricles can be commonly observed,^50^ which is closely related to the symptoms of cognition and gait disturbance.^51^ Therefore, we further investigated the role of increased Neu5Ac in the long‐term preservation of white matter integrity using MRI and brain pathology. Using MRI, we observed that kaolin injection significantly increased the Evans' ratio and PVM hyperintensities in mice (Figure 5A–C, *p* < 0.05). ManNAc reduced PVM hyperintensities caused by hydrocephalus (Figure 5C, *p* < 0.001). However, it failed to prevent dilation of the lateral ventricles in the hydrocephalic mice (Figure 5B).

Periventricular white matter hyperintensities could indicate the presence of demyelination in the pathology.^52^ MBP immunostaining was further examined (Figure 5D,E). Hydrocephalus led to extensive demyelination in the PVM, presenting as a reduction in MBP fluorescence intensities in the corpus callosum (CC), cingulum bundle (CG), and external capsule (EC, Figure 5F, *p* < 0.01). ManNAc effectively augmented MBP fluorescence intensity in the CG (Figure 5F, *p* < 0.05) and EC (Figure 5F, *p* < 0.01). However, the similar trend observed in the CC region (Figure 5F) was not statistically significant.

Consistent with the results of MBP staining, hydrocephalus induced a profound decrease in the intensity of LFB staining (Figure 5G,H, *p* < 0.001) in the CC and EC regions, representing a marked demyelination.^53^ Simultaneously, the intensity of LFB in the EC and CC regions increased in the HCP + ManNAc group compared with that in the HCP + Veh group (Figure 5H, *p* < 0.05). These data suggest that long‐term hydrocephalus‐induced demyelination can be alleviated by increasing Neu5Ac levels in the brain.

### 3.7 Increasing Neu5Ac by ManNAc administration ameliorates neurological deficits in hydrocephalic mice

**FIGURE 6:**
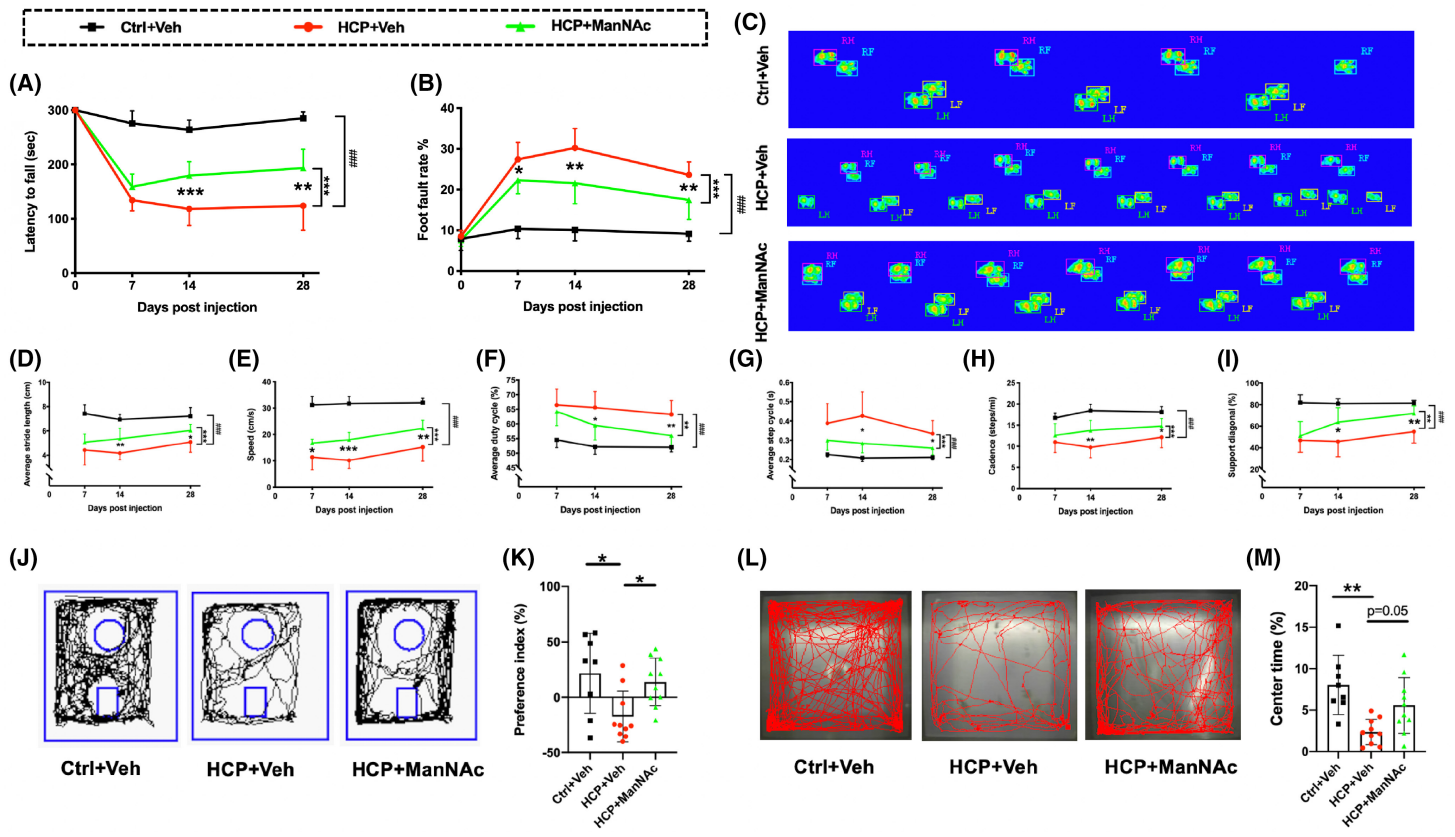
Increasing brain Neu5Ac alleviates hydrocephalus‐induced neurological deficits. (A) The balance ability was evaluated using the rotarod test. (B) The motor coordination was assessed using the foot‐fault test. Data were compared by repeated‐measures ANOVA and post hoc Tukey multiple comparisons. (C) Representative footprints from animals on day 28. (D‐I) Gait parameters, including average stride length (D), speed (E), average duty cycle (F), average step cycle (G), cadence (H), and support diagonal (I), analyzed using the CatWalk system. Data were compared by repeated‐measures ANOVA and post hoc Tukey multiple comparisons. For A‐I, ***p < 0.001; **p < 0.01; *p < 0.05 for HCP + Veh vs. HCP + ManNAc; ###*p <* 0.001 for Ctrl+Veh vs. HCP + Veh. (J) Representative traces of animals during the test session of the novel object recognition test starting from day 28. (K) The preference index was calculated from the novel object recognition test. Data were compared by Kruskal–Wallis with the post hoc Dunn test. (L) Representative traces of animals during the open field test on day 14. (M) The time spent in the central zone during the open field test on day 14. Data were compared by one‐way ANOVA with post hoc Tukey–Kramer test; For J‐M, **p < 0.01; *p < 0.05. For A‐M, *n* = 8 for Ctrl+Veh: animal control + saline vehicle; *n* = 10 for HCP + Veh: hydrocephalic mice + saline vehicle, HCP + ManNAc: hydrocephalic mice + ManNAc treatment. All data were presented as mean ± SD.

We assessed the effect of increasing brain Neu5Ac levels on hydrocephalus‐induced neurological deficits using a battery of behavioral tests. Deteriorated balance ability and motor coordination in hydrocephalic mice were observed using the rotarod and foot‐fault tests (Figure 6A,B, *p* < 0.001). ManNAc markedly increased the running time on the rotating rod from days 14–28 (Figure 6A, *p* < 0.01) and lowered the foot‐fault rate of forelimbs from days 7–28 (Figure 6B, *p* < 0.05). These results demonstrate that increasing brain Neu5Ac levels by ManNAc could help improve motor coordination and balance ability.

Similar to the characteristics of NPH patients,^47^ gait analysis revealed decreased stride length and hypokinetic and dysregulated dynamic equilibrium in hydrocephalic mice (Figure 6C). However, stride length increased in the group of HCP + ManNAc from days 14–28 (Figure 6D, *p* < 0.05). The hypokinetic characteristics were also improved by ManNAc administration (Figure 6E–H). This decreased speed was restored by ManNAc administration at every testing time point (Figure 6E, *p* < 0.05). Other parameters, including average duty cycle, step cycle, and cadence, improved on days 14 and 28 (Figure 6F–H, *p* < 0.05). In addition, on days 14 and 28, diagonal support, assessing dynamic equilibrium, increased significantly after ManNAc administration in hydrocephalic mice (Figure 6I, *p* < 0.05).

The learning and memory function in long‐term recovery was assessed in animal models via the NOR test starting from day 28 (Figure 6J). A significantly lower value of PI was found in the group of HCP + Veh compared to that in the Ctr + Veh group (Figure 6K, *p* < 0.05), demonstrating cognitive dysfunction. Increasing Neu5Ac by ManNAc enhanced the ability to remember the familiar object and recognize the novel object in hydrocephalic mice, as reflected by the increased PI value (Figure 6K, *p* < 0.05).

The anxiety response was monitored using an open field test on day 14. Hydrocephalic mice treated with saline spent much less time in the central zone than control mice treated with saline (Figure 6L,M, *p* < 0.01). Meanwhile, ManNAc‐treated hydrocephalic mice were relatively more likely to explore the central zone than experimental hydrocephalic mice with saline treatment (Figure 6L,M, *p* = 0.05), suggesting that increasing brain Neu5Ac prevented hydrocephalus‐induced anxiety. Altogether, these results demonstrate that hydrocephalus‐induced neurological deficits can be mitigated by increasing Neu5Ac levels in the brain.

## 4 DISCUSSION

This metabolomic study aimed to explore the metabolic profiles and identify the metabolites associated with neurological deficits in NPH. Herein, we demonstrated that three metabolites differed between patients with NPH and controls. Only decreased Neu5Ac was correlated with severity‐related clinical parameters in NPH patients, suggesting its potential role in neurological deficits in NPH. We established a kaolin‐induced hydrocephalic mouse model. ManNAc not only increased brain Neu5Ac in hydrocephalic mice but also inhibited astrogliosis, promoted A1 to A2 astrocyte polarization and attenuated the demyelination of the PVM. We further demonstrated that ManNAc alleviated hydrocephalus‐induced neurological deficits, including loss of balance and coordination, gait disturbance, anxiety, and cognitive dysfunction. Our study provides a novel therapeutic target for NPH.

Utilizing CSF samples from patients with NPH and controls, we identified 12 significantly differential metabolites in the discovery cohort, but only three of them (Neu5Ac, hypoxanthine, and creatine) were found to differ significantly in the validation cohort after adjusting for age and gender. The other nine metabolites, such as glyceric acid, were not significantly different between the two groups in the validation cohort. It should be noted that all three differential metabolites have been reported to change significantly in patients with NPH^18^, ^54^, ^55^ and are correlated with Evans' ratio, which measures NPH‐induced ventricular enlargement. These results indicate the reliability of our metabolomic study.

Although the role of these metabolites in hydrocephalus‐induced neurological deficits remains unclear, we chose to focus on Neu5Ac for subsequent experiments for two reasons. First, its levels were negatively correlated with severity‐related parameters, such as total NPHGS scores and scores of gait and urinary domain. Second, Neu5Ac was the best metabolic factor for differentiating NPH patients in both multivariate and univariate analyses.

We speculated that decreased CSF levels of Neu5Ac could reflect decreased brain Neu5Ac levels caused by hydrocephalus‐induced hypoxia.^23^ The synthesis of brain Neu5Ac is impaired under hypoxia.^56^ For compensation, less Neu5Ac was released into the CSF, and more Neu5Ac in the CSF could be incorporated into cells.^57^, ^58^ Consistent with our speculation, decreased Neu5Ac levels were detected in hydrocephalic mice.

To increase brain Neu5Ac levels, we administered ManNAc, the physiological precursor of Neu5Ac, which is commonly used to enhance sialylation and Neu5Ac levels in the brain.^59^, ^60^ It can enter the brain across the blood–brain barrier and convert into Neu5Ac.^61^ Additionally, ManNAc could sustainably increase the production of free Neu5Ac in the blood, which could then be transferred into the brain across the blood–brain barrier.^60^, ^62^ ManNAc is beneficial in treating various Neu5Ac‐deficient diseases, including GNE myopathy, has been found to improve symptoms related to motor and cognitive functions in aging mice.^41^, ^42^ Thus, we evaluated the efficacy of ManNAc in increasing brain Neu5Ac levels and ameliorating hydrocephalus‐induced pathology and neurological deficits. We conducted a dose gradient study to determine the optimal dose of ManNAc and administered it subcutaneously, as it has no first‐pass effect and a relatively long retention time according to its pharmacological characteristics and previous reports.^39^, ^40^

In the hydrocephalic mice, we observed astrocyte activation and A1 astrocyte polarization in the white matter of hydrocephalic mice. Increasing brain Neu5Ac ameliorated astrogliosis and promoted A1 to A2 astrocyte polarization. Reactive astrocytes are critical modulators of CNS injury, especially in demyelination pathology.^63^ Being phenotyped into A1 and A2 astrocytes, A1 astrocytes reportedly worsen white matter lesions,^64^ whereas A2 astrocytes can promote the repair of white matter.^24^ Therefore, we evaluated the effect of increasing Neu5Ac levels on white matter lesions.

Chronic white matter lesions were observed in the periventricular region of hydrocephalic mice using MRI and pathology techniques, which included MBP and LFB immunostaining to assess demyelination. Increasing brain Neu5Ac levels led to a reduction in white matter hyperintensities on MRI in the PWM of hydrocephalic mice. This was accompanied by a decrease in myelin loss on histological examination. In pathological conditions, chronic demyelination is often accompanied by spontaneous remyelination,^65^, ^66^ which is a crucial process in restoring the myelin structure, alleviating irreversible nerve degeneration, and facilitating the recovery of neurological functions.^67^, ^68^ The remyelination process requires the clearance of myelin debris by glial cells, which further promotes the differentiation of oligodendrocyte precursor cells (OPC) and new myelin sheath extension.^69^, ^70^ During the process of demyelination and remyelination, astrocytes are activated immediately and polarized within the pathological foci.^71^ These polarized astrocytes can be either damaging or beneficial. A1 astrocytes have been reported to impede remyelination.^64^ They lose the ability to phagocytize myelin debris^69^ and decrease microglial phagocytosis via the astrocytic C3‐microglial C3a‐receptor axis.^70^ Furthermore, A1 astrocytes can release neurotoxins such as tumor necrosis factor‐α (TNF‐α), D‐serine, and nitrogen monoxide to form a proinflammatory milieu and kill matured oligodendrocytes directly.^24^, ^72^ In contrast, A2 astrocytes promote remyelination by facilitating OPC maturation through the secretion of neurotrophic factors, such as transforming growth factor‐β (TGF‐β) and brain‐derived neurotrophic factor (BDNF).^25^, ^68^

Considering the changes in astrocyte phenotypes and altered states of myelination in our study, we propose that A1 astrocytes may participate in the demyelination process and prevent spontaneous remyelination in hydrocephalic mice.^64^ Inhibition of astrocyte activation and their transition from A1 to A2 polarization by increasing brain Neu5Ac possibly contributes to the mitigation of demyelination in hydrocephalic mice. In the future, in vitro co‐culture systems combining activated astrocytes and other myelin‐related cells may be used to validate this hypothesis.

In patients with NPH and hydrocephalic mice, demyelination in the PWM, not the size of the cerebral ventricle, was believed to be significantly associated with motor and cognitive deficits.^50^, ^51^ Therefore, we evaluated hydrocephalus‐induced neurological deficits and the effects of Neu5Ac. Along with the alleviation of demyelination, the neurological deficits in hydrocephalic mice, including the loss of balance and coordination, gait disturbance, anxiety, and cognitive dysfunction, improved significantly after increasing brain Neu5Ac. These findings further identified Neu5Ac as a potential therapeutic target for NPH treatment.

The reduction of brain Neu5Ac has been associated with demyelination and neurological deficits in animal models. In humans, biallelic mutations in the NANS gene,^73^ which impairs endogenous Neu5Ac synthesis, have been linked to prenatal hydrocephalus and demyelination.^60^, ^74^ Experimental studies have shown that low‐dose neuraminidase‐induced astrogliosis and demyelination,^75^, ^76^ while high‐dose neuraminidase‐induced hydrocephalus in rats.^21^ However, there is currently no commercialized pharmacological intervention available to reduce brain Neu5Ac in animal models. There are gene‐editing animal models targeting endogenous Neu5Ac production being developed recently.^77^, ^78^, ^79^ In some of these models, brain Neu5Ac decreases to varying extents and impaired motor function could be observed.^77^, ^80^ Nonetheless, these models have not been well studied with regard to demyelination pathology. Developing more consistent and reliable approaches to reduce brain Neu5Ac could aid in further understanding its role in demyelination and neurological injuries.

The mechanism by which increased Neu5Ac levels regulate astrocyte activation and polarization remains unclear. We speculated that this might be achieved by elevating the polysialic acid‐neural cell adhesion molecule (PSA‐NCAM) content. ManNAc can increase the PSA‐NCAM content both in vitro and in vivo.^59^, ^81^ Increasing the expression of PSA‐NCAM in astrocytes has a protective effect on CNS injuries by promoting neural precursor cell migration.^82^, ^83^ However, future studies must address whether increased PSA‐NCAM levels in astrocytes could facilitate A1 to A2 astrocyte polarization.

Our study had some limitations. First, we could not obtain CSF samples from healthy controls in the metabolomic analysis owing to ethical concerns. Therefore, we collected CSF samples from patients having inflammatory demyelinating diseases with pathophysiologies different from those of NPH. Consequently, the two groups were not age‐ and sex‐matched. However, we eliminated confounding effects using statistical analysis. Meanwhile, exploring the association between metabolites and NPH‐induced neurological impairments did not require control data. Therefore, our finding that Neu5Ac plays a vital role in NPH‐induced neurological deficits is reliable. Second, the relatively small sample size used in the metabolomic analysis may have limited the ability to detect all potential metabolic changes that could be present in the broader population of patients with NPH. Nevertheless, we ensured that the sample size met the minimum requirement with high statistical power as per the formula for determining the minimum sample size in a cross‐sectional study.^48^ It should also be noted that NPH is a relatively rare disease, and obtaining a larger sample size would have been challenging due to the invasive nature of the lumbar puncture required for our analysis. For future research, we intend to overcome this limitation by expanding our patient recruitment to include a more diverse population from multiple medical centers. Third, another potential limitation of our study is that we utilized only one mouse model of hydrocephalus induced by kaolin injection into the cisterna magna. To date, there are a variety of experimental models available to study hydrocephalus, each with their own strengths and limitations.^84^ However, no animal model perfectly replicates NPH. We chose this model because it is reliable, well‐established, and highly reproducible.^49^ More importantly, it also resembles human NPH in metabolic disturbance, brain imaging, clinical symptoms, and pathology. It would be valuable in future research to validate our findings and therapeutic approaches in other animal models of hydrocephalus. Furthermore, it would be beneficial for future studies to conduct a comprehensive pharmacokinetic analysis of ManNAc in hydrocephalic mice. This could aid in the clinical translation of the results. Specifically, examining the retention time and bioavailability of ManNAc in hydrocephalic mice using various administration routes would be informative.

In conclusion, our results demonstrated that decreased CSF levels of Neu5Ac were associated with overall clinical symptoms, gait symptoms, and urinary symptoms of human NPH. Decreased brain Neu5Ac levels were observed in hydrocephalic mice. Increasing brain Neu5Ac levels by ManNAc administration suppressed astrogliosis, promoted A1 to A2 astrocyte polarization, reduced demyelination, and alleviated hydrocephalus‐induced neurological deficits. Our study expands the current understanding of how Neu5Ac affects hydrocephalus‐induced neurological deficits. Increasing brain Neu5Ac levels may be a novel therapeutic strategy for NPH.

## AUTHOR CONTRIBUTIONS

Jing Ding and Xin Wang designed the study. Zhangyang Wang, Xiaoqun Nie, Fang Gao, and Yiying Zhang performed the experiments. Yanmin Tang provided help with data analyses. Zhangyang Wang and Xiaoqun Nie drafted the manuscript. Yanqin Gao, Chen Yang, Jing Ding, and Xin Wang reviewed and provided advice for this manuscript. All authors read and approved the final manuscript.

## CONFLICT OF INTEREST STATEMENT

The authors report no competing interests.

## Supporting information


Data S1.

Table S1.
Table S2.
Table S3.

## Data Availability

The data that support the findings of this study are available from the corresponding author upon reasonable request.
