# Supplementary figures and images for: Increasing brain N‐acetylneuraminic acid alleviates hydrocephalus‐induced neurological deficits
Source: CNS Neurosci Ther. 2023 May 24;29(11):3183–98. doi: 10.1111/cns.14253 (PMC10580356; doi:10.1111/cns.14253)

# Full unedited blots for Figure 4

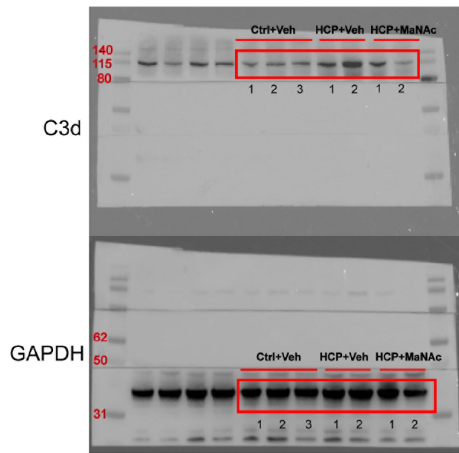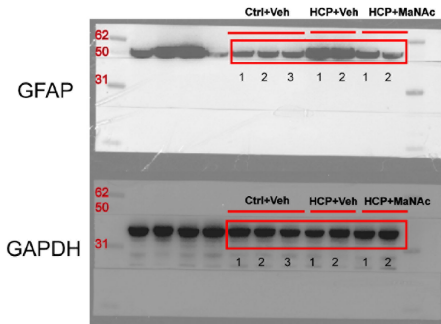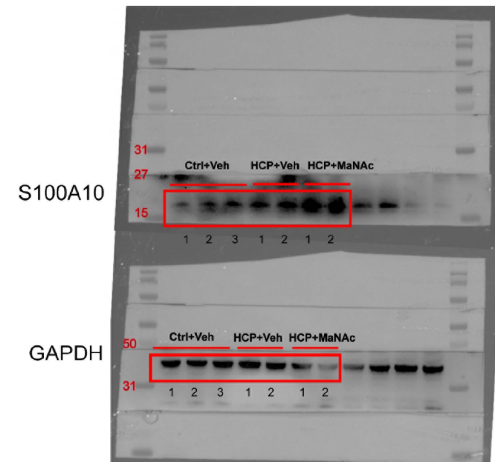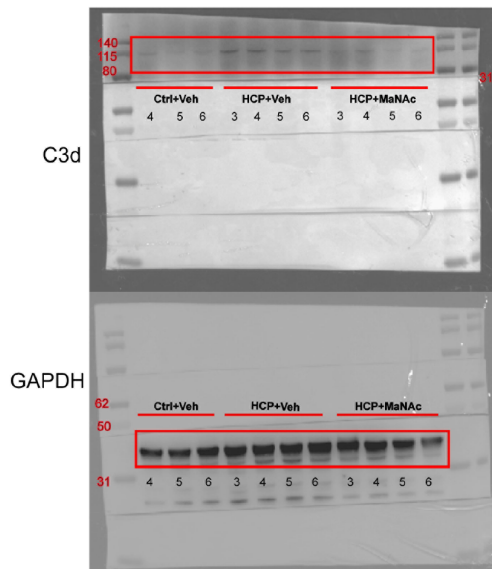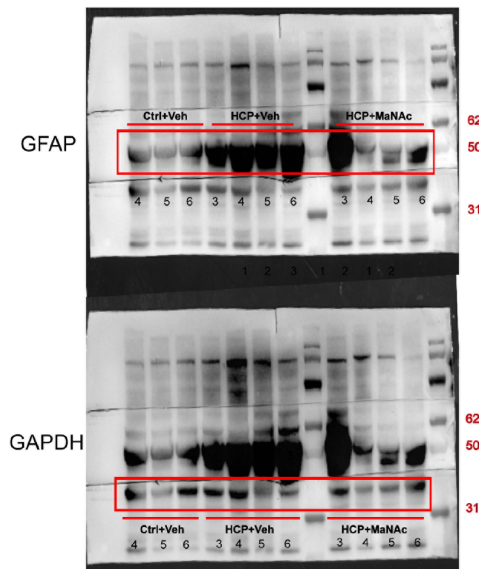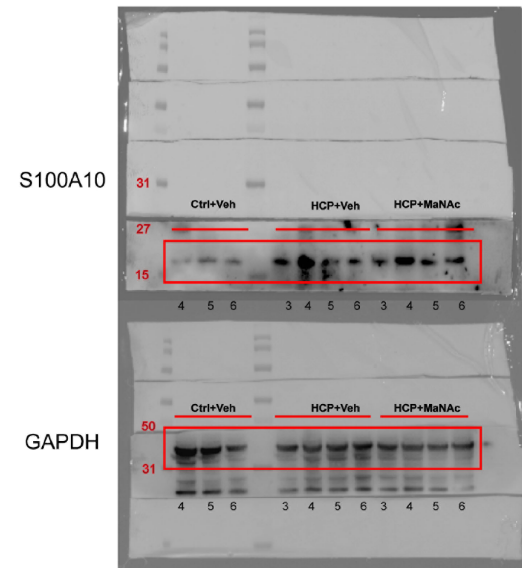

Supplement: Supplementary file 1 — Data S1. [file CNS-29-3183-s002.pdf]
