# Supplementary material for: Increasing brain N‐acetylneuraminic acid alleviates hydrocephalus‐induced neurological deficits
Source: CNS Neurosci Ther. 2023 May 24;29(11):3183–98. doi: 10.1111/cns.14253 (PMC10580356; doi:10.1111/cns.14253)
Supplement: Supplementary file 2 — Table S1. Table S2. Table S3. [file CNS-29-3183-s001.docx]

**SUPPLEMENTARY TABLE 1 Demographics and clinical characteristics of iNPH and sNPH groups**

|  | iNPH | sNPH | *P* value |
| --- | --- | --- | --- |
| Total Number^‡^ | 33 | 9 |  |
| Age^†^ | 72.03±9.07 | 69.44±13.67 | 0.79 |
| Gender (Female/Male) ^‡^ | 9/24 | 1/8 | 0.58 |
| Etiology^‡^ | Idiopathic | CVD:5; Trauma:2；Tumor:1；AS: 1 |  |
| Evans’ Ratio^†^ | 0.33±0.07 | 0.33±0.04 | 0.16 |
| Triad of symptoms |  |  |  |
| Gait disturbance^‡^ | 32/33 | 8/9 | 0.31 |
| Cognitive impairment^‡^ | 32/33 | 9/9 | 0.60 |
| Urinary incontinence^‡^ | 19/33 | 6/9 | 0.62 |
| Complete triad^‡^ | 20/33 | 6/9 | 0.74 |
| NPHGS Scores | 5.88±2.20 | 6.22±2.11 | 0.68 |
| Gait domain^†^ | 2.21±0.93 | 2.33±1.23 | 0.49 |
| Cognitive domain^†^ | 2.61±0.86 | 2.78±0.44 | 0.45 |
| Urinary domain^†^ | 1.06±1.17 | 1.11±1.05 | 0.77 |
| mRs Scores^†^ | 2.52±0.97 | 2.67±0.87 | 0.74 |
| ICP (mmH2O) ^†^ | 142.88±34.65 | 124.29±35.99 | 0.21 |
| CSF analysis |  |  |  |
| Protein (g/L) ^†^ | 0.44±0.15 | 0.46±0.18 | 0.99 |
| Glucose (mmol/L) ^†^ | 3.93±0.84 | 3.91±0.55 | 0.68 |
| Chloride (mmol/L) ^†^ | 125.33±4.20 | 123.64±3.94 | 0.28 |
| LDH (U/L) ^†^ | 16.66±6.57 | 21.22±9.34 | 0.10 |

Abbreviations: iNPH: Idiopathic normal pressure hydrocephalus; sNPH: Secondary normal pressure hydrocephalus; CVD: Cerebrovascular Disease; AS: Aqueduct stenosis;mRs: Modified Rankin Scale; NPHGS: Normal pressure hydrocephalus grading scale; ICP: Intracranial pressure; CSF: Cerebrospinal fluid; LDH: Lactate dehydrogenase

^†^Continuous variables are expressed as median±SD.

^‡^Categorical variables are expressed as n.

**SUPPLEMENTARY TABLE 2 Differential metabolites between NPH patients and controls in the discovery cohort**

| Metabolite | *p* Value | FDR | VIP | Log2 fold-change^†^ |
| --- | --- | --- | --- | --- |
| Glutamic acid | 8.60E-08 | 1.02E-05 | 2.3 | -1.2 |
| Homoserine | 2.44E-07 | 1.93E-05 | 2.13 | 1.3 |
| Glyceric acid | 3.43E-07 | 2.77E-05 | 2.74 | -1.96 |
| Creatinine | 6.68E-07 | 4.10E-05 | 1.86 | 0.43 |
| Linoleic acid | 6.10E-06 | 2.66E-04 | 1.09 | -1.14 |
| N-Acetylneuraminic acid | 8.23E-06 | 2.79E-04 | 2.27 | -3.34 |
| Glycolic acid | 8.37E-04 | 0.01 | 1.68 | -0.21 |
| Tryptophan | 1.39E-03 | 0.01 | 1.33 | -0.47 |
| Creatine | 2.52E-03 | 0.02 | 1.54 | -0.29 |
| Hypoxanthine | 4.02E-03 | 0.03 | 1.11 | 0.39 |
| Quinic acid | 4.23E-03 | 0.05 | 1.28 | 0.72 |
| Itaconic acid | 0.01 | 0.04 | 1.13 | 0.39 |

Abbreviations: FDR: false discovery rate; VIP: variable importance in projection

^†^Log2 fold-change: NPH VS Controls.

**SUPPLEMENTARY TABLE 3 Correlation of 3 differential metabolites levels with clinical parameters in NPH patients**

|  | N-Acetylneuraminic acid | | Hypoxanthine | | Creatine | |
| --- | --- | --- | --- | --- | --- | --- |
|  | r | *P* | r | *P* | r | *P* |
| Evans’ Ratio | -0.46 | <0.001 | 0.42 | <0.001 | -0.32 | 0.004 |
| NPHGS Scores | -0.35 | 0.03 | -0.27 | 0.08 | 0.02 | 0.899 |
| Cognitive domain | 0.02 | 0.83 | 0.11 | 0.49 | 0.03 | 0.84 |
| Gait domain | -0.31 | 0.04 | -0.10 | 0.56 | 0.12 | 0.45 |
| Urinary domain | -0.30 | 0.05 | -0.37 | 0.03 | -0.04 | 0.83 |
| mRs Scores | -0.29 | 0.07 | 0.06 | 0.70 | 0.01 | 0.99 |

Abbreviations: r: correlation coefficient
